# Solid waste generation prediction model framework using socioeconomic and demographic factors with real-time MSW collection data

**DOI:** 10.1177/0734242X241231414

**Published:** 2024-02-26

**Authors:** Laurie Fontaine, Robert Legros, Jean-Marc Frayret

**Affiliations:** 1 Department of Chemical Engineering, Polytechnique Montreal, Montreal, Canada; 2 Department of Mathematics and Industrial Engineering, Polytechnique Montreal, Montreal, Canada

**Keywords:** Municipal solid waste, agent-based simulation models, waste prediction, GIS environment, household behaviours, end-of-life product flows, socioeconomic and demographic factors

## Abstract

This article proposes a framework for developing predictive models of end-of-life product flows, highlighting the importance of conducting thorough analyses before developing waste management and end-of-life product flow strategies. The framework emphasizes the importance of recognizing the nature and quality of the available data and finding a balance between model development time and detail requirements. It is designed to adapt to source material heterogeneity and address varying data availability scenarios, such as the presence or absence of radio frequency identification chips. A case study for the city of Gatineau is presented, showcasing the framework’s application through agent-based simulation models in a geographic information systems environment. The study focuses on creating models of municipal solid waste generation based on socioeconomic and demographic factors and collection data to accurately predict the quantity and quality of waste streams, enabling municipalities to assess the environmental impact of their waste management strategies.

## Introduction

In the context of a circular economy, waste management is gaining new importance regarding resources to be exploited sustainably. Collectively Canadian households send 10 million tonnes of waste annually to landfills, or about 460 kg per person per year (Statistique Canada, 2019). Although landfilling has long been the only way to dispose of residual materials, municipalities now face a challenge in adhering to the 3R-RD hierarchy through their solid waste management system, that is, Reduce, Reuse, Recycle, Reclaim and, if not possible, Dispose of these materials.

The amount and composition of solid waste generated must be known in order to operate, plan and optimize a solid waste management system more efficiently. The heterogeneity in quantity and quality of the material flows generated complicates its treatment and hinders its recovery (Sharma et al., 2019). Furthermore, many factors, such as demographics, incomes and individual behaviours, affect municipal solid waste (MSW) generation (Ceylan, 2020). Several recent studies have argued that a management system analysis should include both environmental as well as financial and social elements (Weng and Fujiwara, 2011), thereby fully adhering to sustainability objectives.

This article aims to provide a framework for reliably using the knowledge discovery in databases (KDD) process with MSW data. This framework will assist scientists in efficiently designing the KDD process stage and dealing with missing MSW data, producing knowledge with higher quality in our field.

## Literature review

MSW generation models usually predict quantity and quality of MSW in order to optimize and plan collection and treatment operations and capacities. The literature proposes several models and studies to forecast future waste generation patterns and to evaluate the impact of different waste management strategies. They are also useful in identifying trends and patterns in waste generation, which can inform policy and decision-making at the local, regional and national levels. In the following sections, we will explore different types of waste generation models, their components and applications and the challenges associated with their development and use.

### The use of influencing factors in MSW generation models

The influencing factors used in MSW generation models are numerous and diverse. They include demographic factors, such as population and age distribution, as well as socioeconomic factors, such as income levels, education and employment. Dwelling types, waste management practices and industrial and commercial activities can also significantly affect waste generation patterns (Beigl et al., 2008). Seasonal factors, such as holidays and weather conditions, can further influence waste generation patterns (Saladie, 2016). In a study exploring optimal organic material treatment in diverse Mexican cities (De Medina-Salas et al., 2019), waste quantities, moisture content and biochemical methane potential vary significantly across locations. These variations are attributed primarily to factors like socioeconomic strata and dietary patterns, as indicated in their prior research (Castillo-González and De Medina-Salas, 2014). These studies underscore the crucial role of accurate data on MSW generation and composition as a fundamental input for decision-making tools. Furthermore, Xia et al. (2022) sustain that such an influence is not linear and that the local context seems to impact the system’s response to these factors. For example, research by Goel et al. (2017) suggests that an increase in income is associated with higher recycling rates. In contrast, a study conducted by Ferrara and Missios (2005) indicates that a reduction in income may lead to lower recycling rates. Wu et al. (2020) also observed this, using neural networks to analyse and compare the impact of regional factors on model accuracy. The authors have concluded that if some regions share similarities in predictor influence, the use of large-scale models (state or country wide research) should be limited to cities lacking historical data about their own MSW generation.

A significant number of studies successfully represent the generation of one or more collection routes with different level of details (Beigl et al., 2008; Dias et al., 2021; Goel et al., 2017). Collection routes data are typically used to estimate the amount of waste produced from a given area by considering the type, frequency and number of collection services that are available in the area. By using data from collection routes, models can more accurately reflect the varying waste generation rates among different areas and help evaluate the impact of specific waste management strategies, such as changes in collection frequency or the introduction of new collection types or recycling programmes. Additionally, collection routes can help identify areas of waste generation that may require additional attention or resources, such as those with higher waste generation rates.

### Types of MSW generation models

Different types of models are used to predict MSW generation and estimate the quantity and composition of waste in a particular region or community. Common types of MSW generation models are empirical regression models, input–output models, scenario-based models and hybrid models combining different modelling approaches (Edo-Alcón et al., 2016; Kannangara et al., 2018; Xia et al., 2022).

A meta-analysis of MSW generation prediction models (Dias et al., 2021) showed that artificial neural network was the most used technique with 35.8% of the research, followed by linear regression (16%) and support vector machine (12.3%). With the emergence of fields such as data science, several prediction models benefit from hybrid data mining and machine learning mechanisms (Bose and Mahapatra, 2001). These models can therefore observe trends based on historical waste material data.

Despite the performance of machine learning, the black-box quality of these models reduces the interpretability of the relationship between parameters and results (Hoque and Rahman, 2020). In contrast, simulation modelling based on a stochastic and mechanistic approach allows for concrete problem solving based on an awareness of the system in place. This analysis method can be tested and validated while allowing a better understanding of the internal processes. Moreover, uncertainties regarding the waste produced and the population growth under policy change can be included in simulations as done by Huang et al. (2005) and Singh (2019). Simulation can also facilitate communication with stakeholders and enable in-depth comparative analysis to justify investment and planning decisions as it have been done with De Medina-Salas et al.’s (2019) work.

### MSW historical data and model uncertainty

Rapid urban growth and insufficient budgets prevent many municipalities from obtaining complete historical data, limiting the technological tools used, particularly at the household or community level (Ding et al., 2018; Kannangara et al., 2018). Because of this data shortage, Boskovic et al. (2016) identified that containers were the most common collection method used in case studies. However, curbside collection is common in municipalities. Due to the high variability in attributes, such as socioeconomic factors and generation itself, small datasets increase the complexity of the analyses and produce low accuracy models. This highlights the need to invest in model training to demonstrate the value of data structuring and management in the industry (Dias et al., 2021).

As data become more available, geographic information system (GIS) helps to represent interactions in a real system and thus better support the interdependencies between the micro behaviours of the system and the spatial environment (Georgé et al., 2003). In waste management simulations, material transport between citizens and treatment centres and land use benefits from GIS and can lead to the optimization of collection routes (Nguyen-Trong et al., 2017; Ruiz-Chavez et al., 2018). The emergence of radio frequency identification (RFID) technology on tags identifying and tracking bin pickup also supports realistic simulations in geographic environments. Imran and Kim (2020) used RFID data, GIS maps waste bins, electronic and predictive data analysis to virtualize waste to its actual location. Smart bin collections were then compared to the daily breakdown of waste in the study area.

Nevertheless, Dias et al.’s (2021) research showed that MSW research’s main challenges are data collection and processing. When forced with insufficient data, Al-Khateeb et al. (2021) recommend tackling this challenge by collecting more information or designing a method suitable for the quantity of data at hand.

A way to design a suitable method with data in mind is the KDD process, which consists of multiple steps such as data selection, preprocessing, data transformation, trend finding (data mining) and visualization for interpretation (Luo, 2008). Working with waste management data requires its transformation through multiple preprocessing steps that must be performed correctly to avoid bias. Bias may come from the variability of individual container weight caused by population’s behaviour and factor mentioned earlier. KDD have been used successfully in MSW studies as presented by Dias et al. (2021) literature review or Korhonen and Kaila (2015) work on MSW data preprocessing to create reliable information on household waste generation. However, this method does not guide the user in case of low-quality data or low availability of data and how to work around these challenges. Therefore, research should support KDD with framework appropriate to MSW particular requirements to avoid interfering with the complexity of scenarios that may be proposed. This framework would need to be flexible towards the datasets and their uncertainty, while helping to position any study in its geographical context and the impact that context may have on the results.

## General methodological framework

This framework aims to propose a general methodology to develop an agent-based simulation model in a GIS environment capable of accurately predicting detailed MSW generation for applications such as collection route and capacity planning.

**Figure 1.**
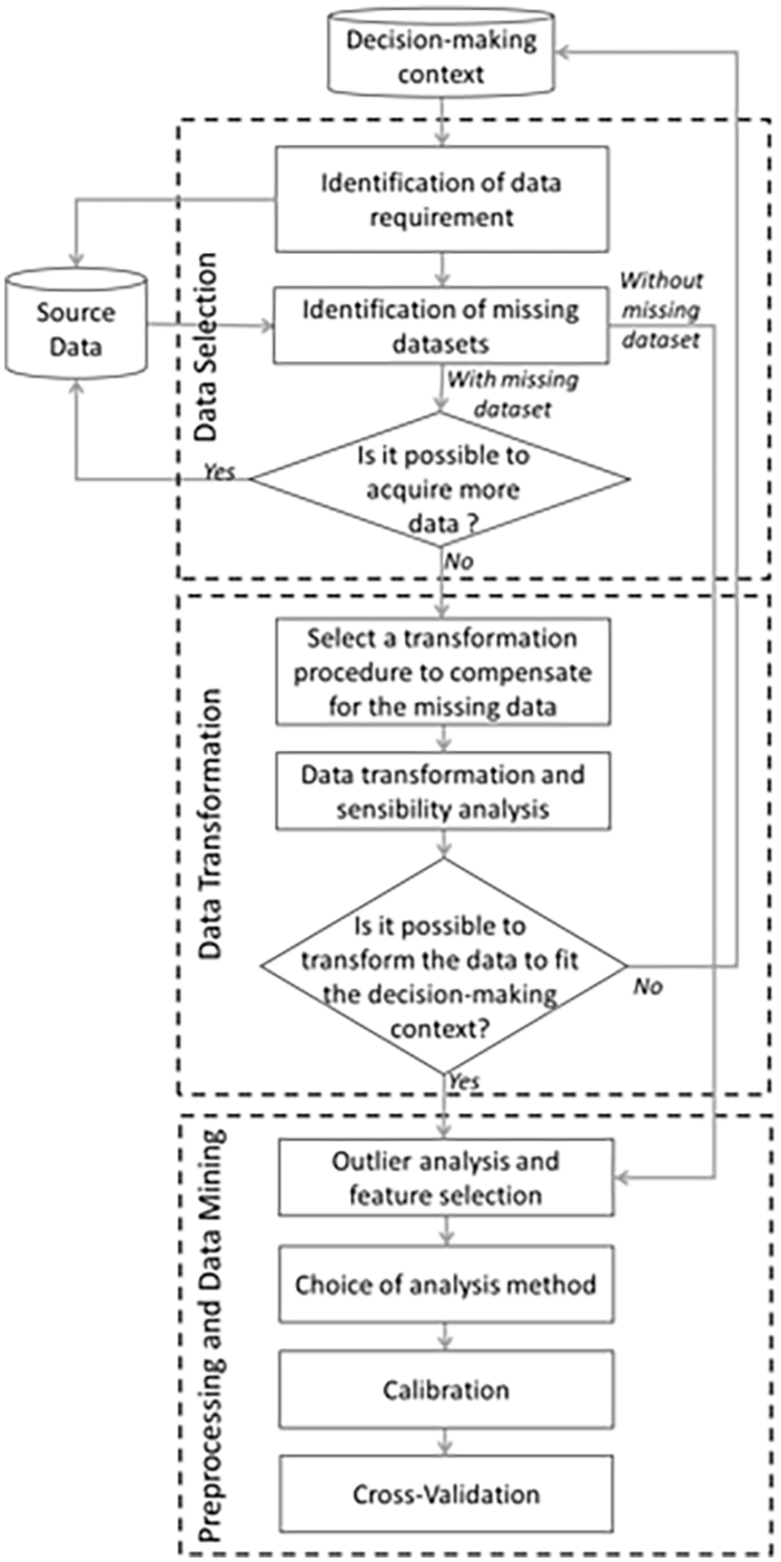
MSW model design framework.

Although the information required from municipalities may vary, the proposed framework aims at building models of dwellings MSW generation based on socioeconomic and demographic factors and possible collection data with a systematic and organized approach. The framework proposed in this study contains three steps as presented in Figure 1: Data Selection, Data transformation and Preprocessing and Data mining.

Firstly, it is necessary to recognize the nature and quality of the data that are available in relation to the decision-making context. For examples, understanding what is known about the dwellings allows the identification of missing datasets to meet the study needs. Without additional information, assumptions based on similar studies or expert opinion, or data transformations, such as adding synthetic data, must be applied. These may decrease the accuracy of a model. In addition, knowing the scope of the study allows to find a balance between model development time and detail requirements.

**Figure 2.**
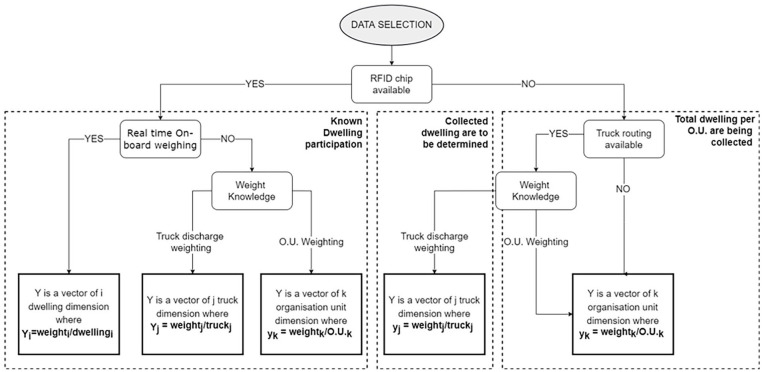
Proposed framework for data selection based in knowledge limit.

Figure 2 presents the proposed general framework for data selection based on knowledge limit. Among others, it shows how to address different cases of data availability, particularly regarding RFID chips access. When RFID chips are present, we have information about the weight per bin, the weight per truck or the weight associated with a specific organizational unit. In this context, organizational unit refers to a distinct and identifiable component or entity within an organization responsible for the efficient and systematic handling of waste-related activities. These units often encompass a variety of entities, such as municipalities, specific geographic regions, fleets of waste collection trucks or segments of truck routing. This changes the generation variability description since the level of details is higher. Then, it is possible to correctly represent citizens’ participation in collective waste collections. Further studies on individual dwelling behaviour are also possible. In cases where RFID chips are not available, if only a global weight is known for the total amount of collected dwellings, the dwelling behaviour is generalized to the entire population, which limits the accuracy of the model.

If intermediate knowledge is obtained, such as the routing of trucks without RFID chips and its total weight, the collected dwellings must be identified for each route. With the emergence of municipal databases, it is possible to track the location of trucks in real time, as well as their material discharge weights during a working day. If waste generation prediction must consider the heterogeneity of the population in terms of sociodemographic profiles and household behaviours, individual addresses must also be tracked to link collected dwelling sociodemographic profiles to the corresponding truck total weight. Although each dwelling cannot be specifically identified (due to lack of individual RFID data), it is still possible to calibrate (i.e. train) a general MSW generation model using dwelling sociodemographic profiles, truck weight and collection route records. To do so, the address dataset must be layered with truck discharge weight and coordinates to deal with the missing RFID data.

Since this scenario requires additional steps, it is the focus of the case study presented in the next section. It demonstrated how the framework can adapt the model development to the source material heterogeneity.

## Application of the proposed research framework: Case of Gatineau’s waste generation

This section aims to apply the proposed methodological framework to the case of an average size Canadian city: Gatineau. Through the case study analysis, we demonstrate the framework’s ability to adapt to the heterogeneity of the source material and address different cases of data selection and transformation. The following sections will detail the methodology and findings of this case study.

### Data selection and case study presentation

The study area chosen is the city of Gatineau, a city adjacent to Canada’s capital, Ottawa, where the population produces on average 8.0 kg dwelling^−1^ week^−1^ of mixed waste that are collected and disposed in landfill by the municipality (Service de l’environnement, 2022). It’s important to note that this figure specifically pertains to mixed waste and does not include data for recycling or organic waste collections. Using a specific city and collection stream for the study ensures that data capture is standard and consistent for each database entry. Choosing mixed waste as a proof of concept not only enhances our understanding of this stream but also lays the foundation for innovative material valorization methods, while also enabling the application of the same methodology to other collection streams in the future. Although RFID technology is being implemented for some MSW collections in the territory, the information was unavailable for this study.

The four databases presented in Table 1 were available for three pre-COVID years. Truck travel records are available for container and bin collections in the territory. It is important to note that most multi-family dwellings are collected by containers, whereas most single-family dwellings are collected by bins. However, this is only true for some dwellings, making it impossible to completely dissociate the two collection types to obtain a complete picture of the territory.

**Table 1.** Gatineau’s available datasets.

	Features	Description
*Trucks travel record*	Truck Identification (ID)	Unique ID of the physical truck
	Time	Recording of the position every 20 seconds
	Latitude	Latitude of the location of the truck
	Longitude	Longitude of the location of the truck
	Speed	Speed of the truck
	Ignition	Status of the motor
*Discharge centre record*	Truck ID	Unique ID of the physical truck
	Hour of discharge	Time of recording
	Waste amount	Weight of waste per truck for 2017 to 2019
*Address database*	Address ID	Unique and anonymous ID for physical addresses
	Latitude	Latitude of the location of the address
	Longitude	Longitude of the location of the address
	Number of dwelling	Number of dwellings at the address
	Type of waste collection	Curbside or container
*2016 Canadian Census data*	Dissemination area (DA) ID	Dissemination area with an average population of 400–700
	DA grid	GIS polygon of the dissemination area
	Population	Person count in the DA
	Dwelling type	Distribution of people per 8 divisions of dwelling type
	Salary	Distribution of people per 11 divisions of salary
	Household size	Mean of household size in the DA
	Schooling	Distribution of people per 3 divisions of diploma
	Age	Distribution of people per 7 divisions of age
	Gender	The male and female population count

GIS: geographic information systems.

For a given collection day, a dwelling is not required to participate in the curbside collection. This non-participation does not mean that waste production is stopped, but rather that the amount may have been adjusted in a subsequent collection. However, according to the law of large numbers, if one repeats an experiment independently a high number of times, the average result should be close to the true value (Hsu and Robbins, 1947), which means that with a sufficient number of dwellings, these inconsistencies become negligible. On the other hand, the strength of the RFID chip is the ability to observe and study non-participation in the collections and thus ensure a better knowledge of the actual number of dwellings collected by a given truck, thereby giving a more accurate value of the distribution of MSW generated per week and per dwelling. In the absence of this information, the challenge is to create a new dataset that links dwellings’ unique ID (i.e. addresses) with trucks’ collect routing, ensuring that it is possible to correctly identify which trucks collect a given dwelling while maintaining a sufficiently large set of dwellings.

**Figure 3.**
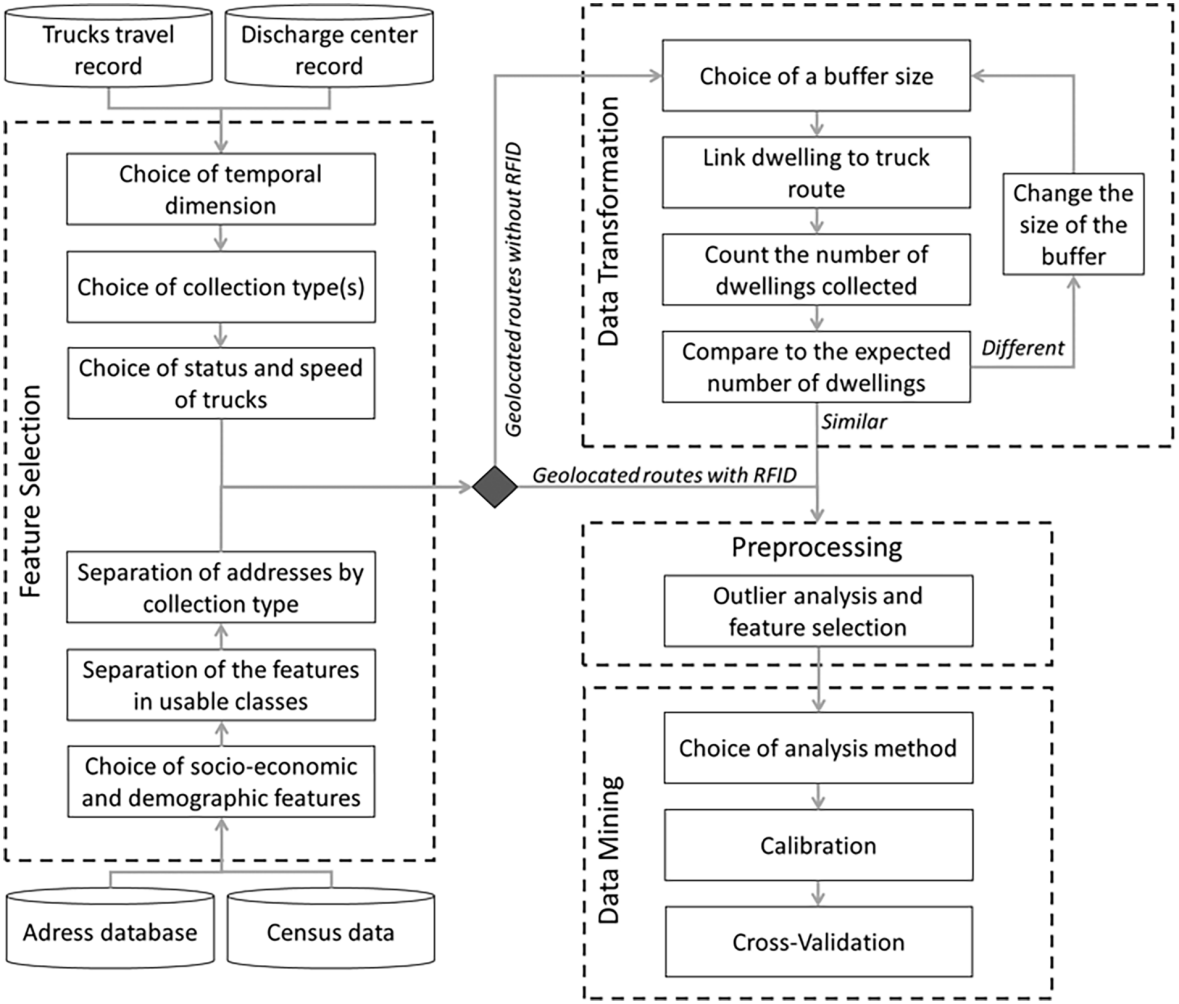
Case study proposed methodology.

The methodology introduced in this section, as depicted in Figure 3, utilizes the overlapping nature of solid waste collection data, which includes information about trucks’ discharged weight and routes. This methodology is employed to develop a predictive model for household solid waste generation, incorporating socioeconomic and demographic factors. Although a set of socioeconomic and demographic parameters is needed to explain MSW generation and the true household behaviour, some are known to have a definite influence on generation, such as housing (Lagneau, 2018). Although the methodology can be applied to many different parameters, housing will be the focus of this study.

Statistics Canada divides dwellings into eight distinct categories, which provide a great deal of information about the area, but do not necessarily correspond to eight different waste generation profiles. Therefore, this study has separated dwellings into two categories: single-family dwellings and the broad category of multi-family dwellings. This allows for focusing on the barriers and waste management challenges often associated with multi-family housing, such as lack of knowledge and education about waste management (Pawlewicz et al., 2019).

### Data transformation and hybridation

Firstly, it is necessary to merge the quantities collected by trucks and their GIS route tracked with GPS. In the meantime, the addresses can be cross-referenced to the polygons described by the Statistics Canada census. Secondly, the lack of RFID information requires the use of a reverse geocoding algorithm to link the collected dwellings to the appropriate truck. This reverse geocoding algorithm aims to match geographic coordinates into human-readable addresses (Karimi and Karimi, 2017). Li et al. (2017) used reverse geocoding to identify evacuation trigger points during a wildfire. A safety buffer around the fire was used to identify homes at risk. Here, we used a similar process to identify potential homes collected by each collection truck at a specific time. To match the GPS coordinates of a truck’s route with addresses, this algorithm uses buffers. A buffer is a circular area defined by a radius and centred at the coordinates of a specific address. If the GPS coordinates of the truck’s route is within the buffer, the dwelling at this address is flagged as collected.

**Figure 4.**
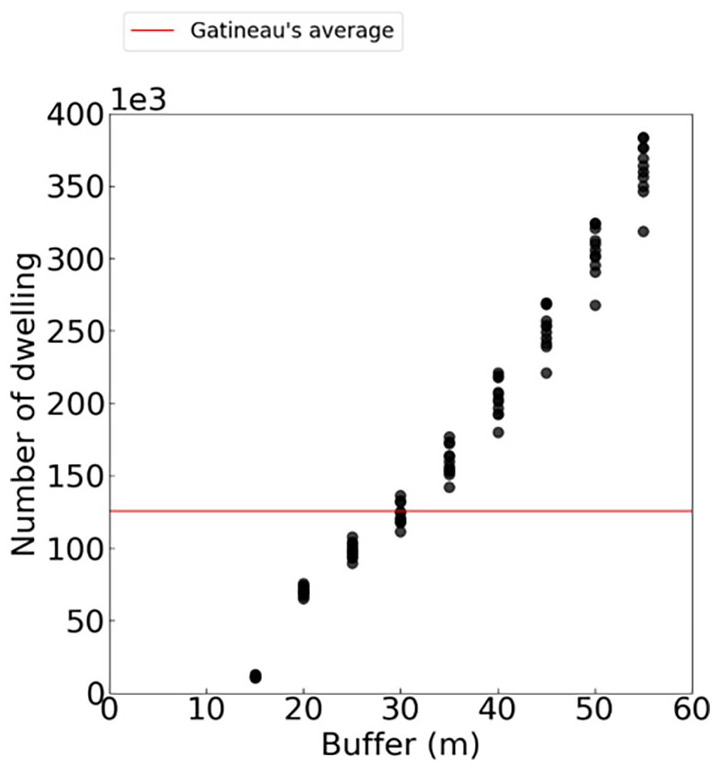
Number of dwellings collected per buffer size.

To adjust the parameters of this algorithm and avoid missing addresses or adding wrong addresses, several buffer sizes (Figure 4) were tested over 10 weeks of waste collection. The 30 m radius buffer appears to provide the closest representation of the actual number of dwellings in the area. This value is interesting for single-family homes in dense areas since 30 m is generally equivalent to the total lot size thus limiting the addition of false positives. Some multi-family dwellings may have their geolocation point further away than 30 m due to the larger size of their building and lot. However, these dwellings are more often collected by containers, bringing the trucks and their buffer closer to the centroids of these buildings.

Since collections in the area can be curbside or by containers, the databases of dwellings and trucks were split so that the correct type of truck could only collect a type of dwelling. The second step is to ensure that the trucks are in collect mode by looking at the engine ignition device and travel speed. Some major roads are frequently used by trucks. Indeed, it is possible to verify that the few homes on these roads were not overrepresented in the new databases by using a maximum collection speed of 15 km hour^−1^. GPS coordinates and speed are not necessarily recorded when the truck stops. However, setting a maximum speed of 15 km hour^−1^ ensures that the truck is most likely in collect mode.

Furthermore, because most databases include missing values and anomalies caused by equipment failure, human error and similar problematic situations (Heilala, 2018), we also addressed outliers and missing data. A mismanagement of these problems can lead to misinterpretations. In waste management, such anomalies may occur for many reasons such as bad weather, a spike in MSW amounts or the collection of wastes outside the typical collection area. Therefore, as described in Figure 3, there is a preprocessing step before data may be analysed.

### Model architecture

As mentioned earlier, this model uses agent modelling and simulation. Each dwelling corresponds to a specific agent with specific attributes such as an address, a type of dwelling and eventually a sociodemographic profile. Each agent randomly generates waste and has specific sorting habits that must be calibrated with collected data. Although it is a rather simple model, the calibration to identify the agents’ waste generation profile and sorting habits is challenging. Every action and decision the agent takes must be calibrated with generation being the focus of this framework. The use of an agent-based simulation model not only facilitates a comprehensive evaluation of waste production but also paves the way for further research into dwelling behaviour.

**Figure 5.**
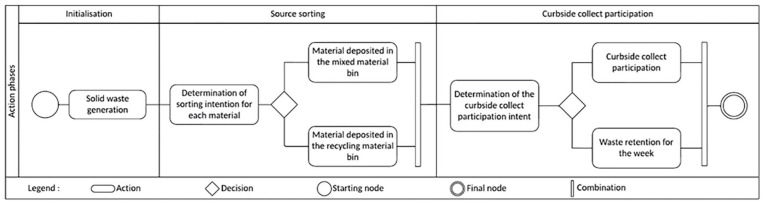
Waste sorting and curbside collect participation state diagram for each modelled week.

The following state diagram dictates each agent’s action (Figure 5). The architecture of the model requires that the generation of materials be identified by dwelling, but also according to key socioeconomic parameters differentiating agents.

### Model implementation and calibration

The model was implemented using the AnyLogic simulation platform. Anylogic is a powerful simulation software that allows users to create complex models and simulations of various systems and processes. Anylogic, while having its own programming language, can also be connected with Python, a popular and flexible programming language with a wide array of libraries and data analysis tools. Additionally, we utilized Python independently from Anylogic for its open-source GIS processing library.

The model calibration methodology uses multiple data sources, including measured truck weights. Several sequential steps were implemented beyond data preparation. Each step helps narrow the experimental conditions to calibrate the model and improve its accuracy. These steps are:

- Select representative weeks using descriptive analysis
- Select a proper training/testing split ratio of trucks’ weights using mean square error (MSE)
- Select outlier elimination process using multi-unit and single-family dwelling generation rates
- Select representative subcategories of the calibrated parameters
- Evaluate the performance of the developed model

The calibration method used is the non-negative least squares solver from the python library SciPy. The optimization is an active set method using a minimization objective function and a set of constraints based on Karush–Kuhn–Tucker conditions that define the feasible region, as published in Lawson and Hanson (1995).

(1) $$\arg\;\underset{\tau }{\min}{\left\|A\tau -b\right\|}^{2}\;\mathrm{for}\;x\geq 0$$

For a given number of trucks (*n*), we represent ‘A’ as a 2-by-n matrix that shows the quantities of waste collected from single-family and multi-family dwellings by each truck. The vector ‘b’ represents the total waste weight in the trucks, and ‘ τ ’ symbolizes the waste generation rate for both single-family and multi-family dwellings in the experiment. Therefore, the performance or the calibrated parameters are used with each truck and their specific population to evaluate the objective function.

An essential part of each calibration is to perform cross-validation, that is, to repeat the experiments several times, removing a different portion of the samples at each repetition. The MSE (equation (2)) was used to assess the capacity of the model to be calibrated, as well as its capacity to make accurate prediction. It is the root mean square difference between the estimated values ( ${\hat{Y}}_{i}$ ) and the actual values ( $Y_{i}$ ).

(2) $$\mathrm{MSE}=\frac{1}{n}\overset{n}{\underset{i=1}{\sum }}{\left(Y_{i}-{\hat{Y}}_{i}\right)}^{2}.$$

## Results analysis

Some truck routes are used to calibrate the model and others to evaluate performance. However, it is possible to do this separation in several different ways:

- Split the collection routes by choosing truck routes independently of their collection days and neighbourhoods
- Split according to the neighbourhoods by combining truck routes
- Split by collection days regardless of neighbourhoods by combining truck routes
- Split by collection days and neighbourhoods by combining truck routes

In order to decide on which separation to perform, it is necessary to know the modelled population. Neighbourhoods in a city can separate the population into similar groups. For example, some neighbourhoods have few multi-dwelling units, whereas others have most of them. This is the case for Gatineau. In addition, each neighbourhood is only collected on certain collection days. By knowing the Gatineau’s territory, the diversity of the population in the dataset is ensured by separating the collection routes independently of the collection days and the neighbourhoods. Therefore, for this specific city all the collection routes were randomly separated.

### Standardization of waste data by selecting data characteristics

**Figure 6.**
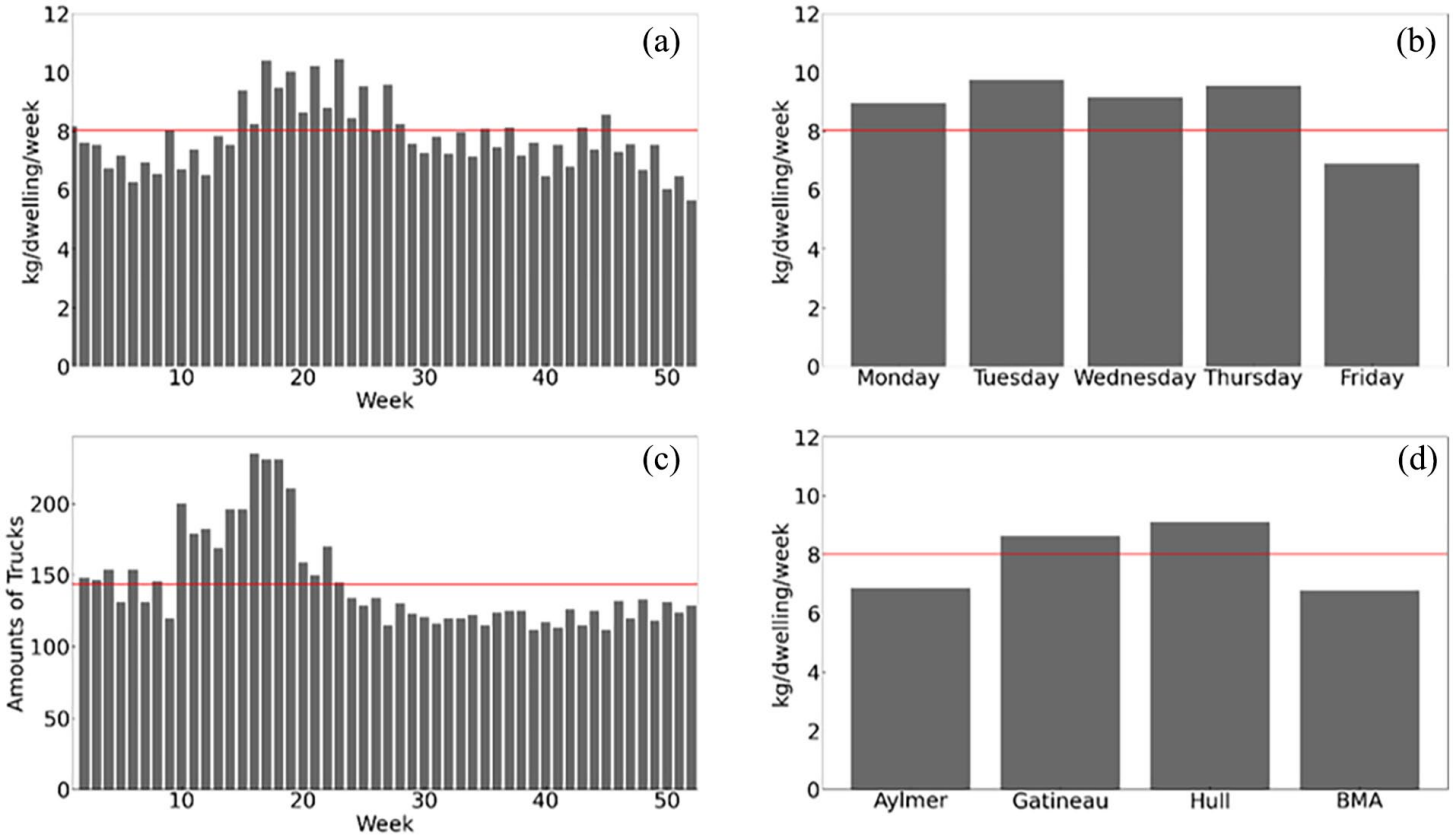
(a) Average waste collected per week per household for a 3-year collection period; (b) average waste collected per week per household per weekday; (c) average amount of trucks per year for a 3-year collection period and (d) average waste collected per week per household per borough.

Data normalization involves converting the database to ensure that the format is consistent and that entries are comparable. As shown in Figure 6, an average of 144 truck routes are recorded each week for the region under study, representing an average of 8.0 kg of waste collected per week per dwelling. However, this weight of material is not constant throughout the year. In the case of waste management, the large variation in MSW generation over the year requires descriptive analysis of the phenomena in order not to discard data that would bias the analysis. The choice of sampling trucks during specific seasons, such as early winter or summer, may lead to an underestimation or overestimation of the annual average waste generation rate and can also affect the composition of the waste stream. This is especially evident when a larger amount of green residues arrive during the summer. In addition, to keep the composition stable and consistent with the city average, specific weeks were selected primarily during the spring season for descriptive analysis.

Furthermore, it can be assumed that the day of the week when a residence is collected does not affect the materials it generates, unlike the collection frequency (Tuckeret al., 2000). However, examining Figure 6(b) and (d) reveals that since different areas are collected on different days, the socioeconomic and demographic characteristics of the dwellings collected can vary, impacting the model’s calibration. To ensure robust calibration and testing, data points should be selected randomly throughout the week.

### Calibration

Since the simulation model does not precisely replicate the waste generation rates and sorting habits of every dwelling, the symbol ‘ τ ’, which represents calibrated waste generation rates, is not a straightforward vector. Instead, it comprises a range of potential waste generation rate values, each calibrated using a specific training dataset.

Sensitivity analyses were performed on the number of trucks included in each training and test split. Therefore, the estimated values ( ${\hat{Y}}_{i}$ ) are the simulated generation rate per truck and the actual values ( $Y_{i}$ ) are the truck collected weight data.

**Figure 7.**
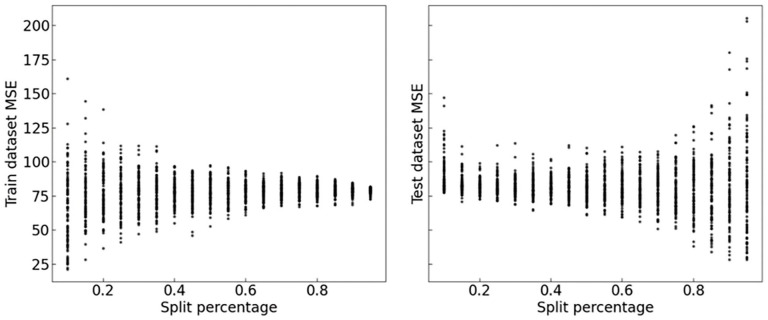
MSE comparison between the train/test split percentages. MSE: mean square error.

Figure 7 shows that by including a larger proportion of trucks during calibration, or training phase, the MSE distribution becomes narrower, likely due to overfitting. On the contrary, during the test phase, the MSE distribution becomes more spread confirming the overfitting hypothesis. Thus, a compromise of 60% of trucks being used in the training set was chosen for each of the 100 repetitions performed.

### Features selection and outlier removal

If a truck can make several discharges in 1 day, its maximum weight capacity at any given time is 15 tonnes, whereas the average discharge weight is 8.4 tonnes. In addition, since trucks can discharge several times per day, an average of 19.9 tonnes of material per truck per day is recorded. However, because we do not know exactly what weight was loaded in each truck, it is reasonable to assume that some datapoints (i.e. truck route and weight) are not representative and should be discarded.

The empirical law, often referred to as the sigma rule, is a statistical rule stating that for a normal distribution 95% of the observed data lie within 2σ of the mean and 99.7% within 3σ. On a bell curve, this technique allows one to classify a value as an outlier if it is outside one of these intervals (Borovcnik, 2007). After having ensured that the collected weights and the number of dwellings were positive and greater than zero, we assessed the impact of interval size criteria on calibration accuracy by testing four conditions: no data cut, a cut at 3σ, a cut at 2.5σ and a cut at 2σ from the mean.

**Figure 8.**
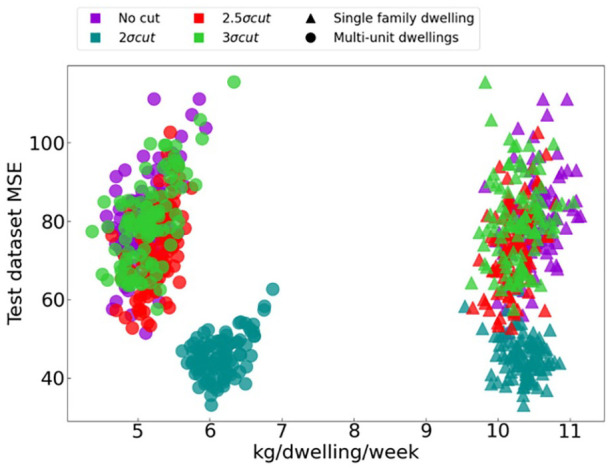
Impact of four sigma rule condition (colour) on the calibration accuracy (MSE) and dwelling type (shape). MSE: mean square error.

In Figure 8, we show the MSE calculated between estimated and actual generation rate values after datapoints judged outliers by the interval sizes were removed. This figure shows that, for all conditions tested, the predicted generation rates for multi-family dwellings are lower than for single-family dwellings although neither is exactly on the average of 8.0 kg dwelling^−1^ week^−1^. In addition, it can be observed that a cut at 2σ from the mean decreases the MSEs of the replicates but increases the amount generated per dwelling per week for multi-family dwellings. All the other cut sizes do not seem to have a clear effect on the MSE.

**Figure 9.**
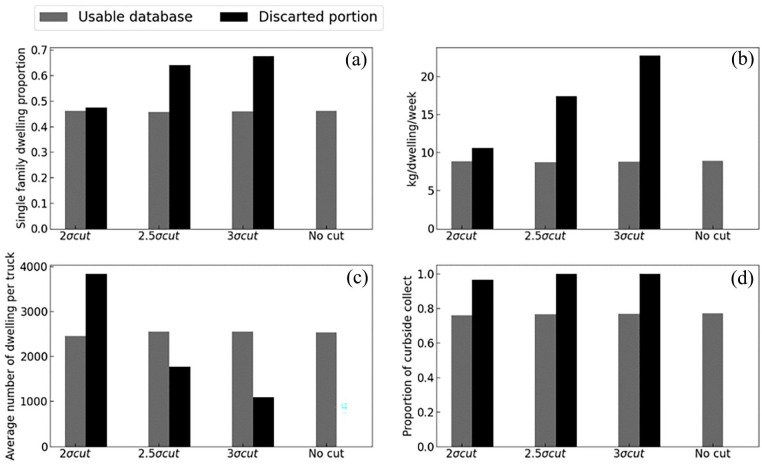
Impact of the sigma rule on the discarded dataset profile for (a) single family dwelling proportion, (b) waste amount per dwelling per week, (c) average number of dwelling per truck and (d) curbside/container collect proportion.

To understand this effect, Figure 9 presents the content of the usable databases as a function of the cut interval sizes. The usable databases generally have an average close to those without discarded data for all parameters presented. Looking at the parameter profiles with the discarded data, as the data are cut, the proportion of single-family dwellings and the rate of material per dwelling per week increases, whereas the average number of dwellings collected per truck decreases.

This suggests that the discarded trucks represent curbside collections with higher-than-expected amount of materials.

However, in Figure 9(c), the 2σ cut has a somewhat different profile with an higher than average number of collected dwelling per truck. Hinting at a population with lower-than-average generation rate. No specific subpopulation must be discarded from the calculation. For instance, if multi-dwellings get discarded by the 2σ cut, leading to an increased MSE, then it is possible that a bias is introduce in the selected dataset. Thus, a cut to a value of 3σ is proposed to ensure the data quality.

### Parameters separation in usable subcategory

**Figure 10.**
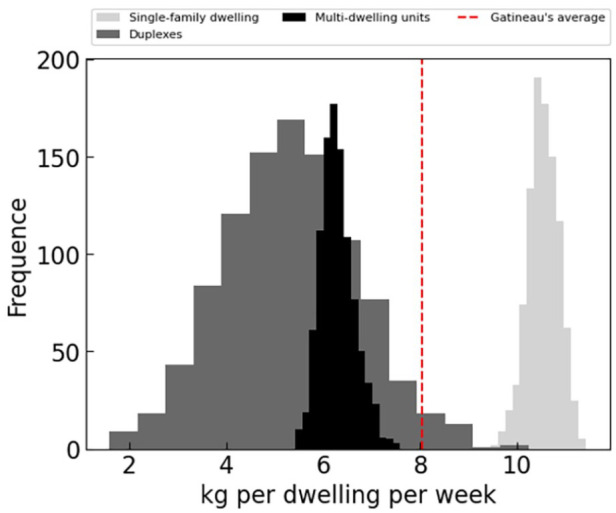
Distribution of waste generation rate per dwelling per week calibrated with three housing types.

The choice of socioeconomic and demographic parameters should not be overlooked, as it can add a source of bias to the model. For example, Quebec municipalities often describes housing as single-family dwellings, duplexes and multi-family dwellings, separating 2–3-unit dwellings that often have access to a courtyard, from buildings with a larger number of units. Although previous studies have shown that for some cities these dwellings have a distinct and traceable behaviour (Lagneau, 2018), this was not observed for our case-study region as shown in Figure 10. The reported data correspond to generation rates calibrated for the three selected housing type. Although results for single-family dwellings are apart from the other 2, results for duplexes overlap those for multi-family dwellings.

**Figure 11.**
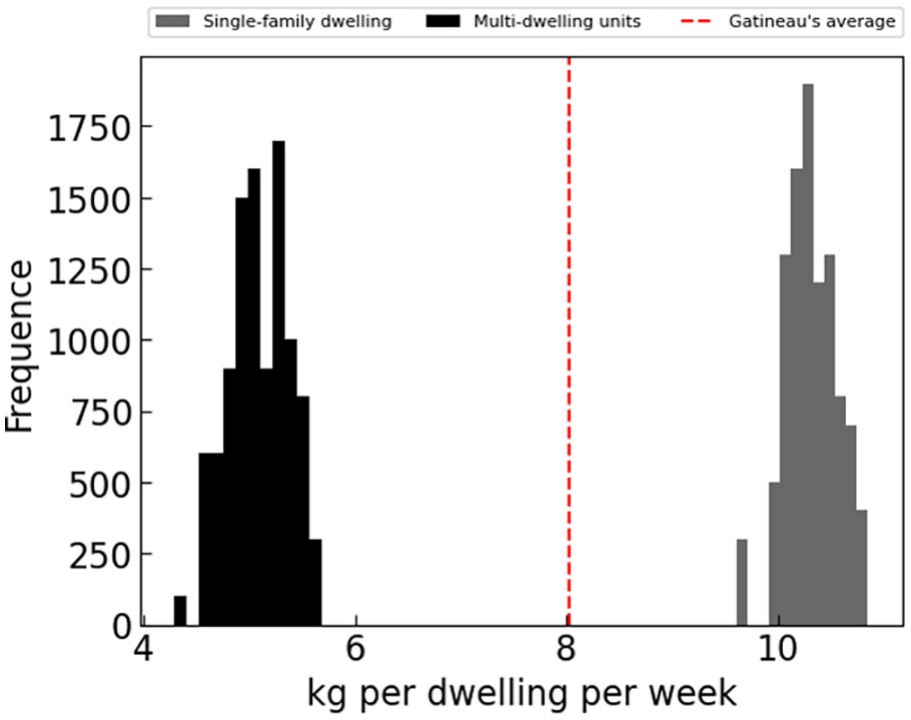
Distribution of waste generation rate per dwelling per week calibrated with two housing types.

The calibration was then performed on only two types of housing: single-family dwellings and multi-family dwellings, with duplexes being considered multi-family dwellings. This calibration led to two distinct behaviours for these two housing-types (Figure 11). The stochastic aspect of the model allowed us to obtain not a single calibrated value, but rather a distribution of possible rates for a housing type. For the agent-based simulations, each agent will then be assigned a personal generation rate from this distribution according to their housing type. With a sufficiently large number of agents, the resulting generation rate distribution should match the calibrated one.

### Performance analysis

**Figure 12.**
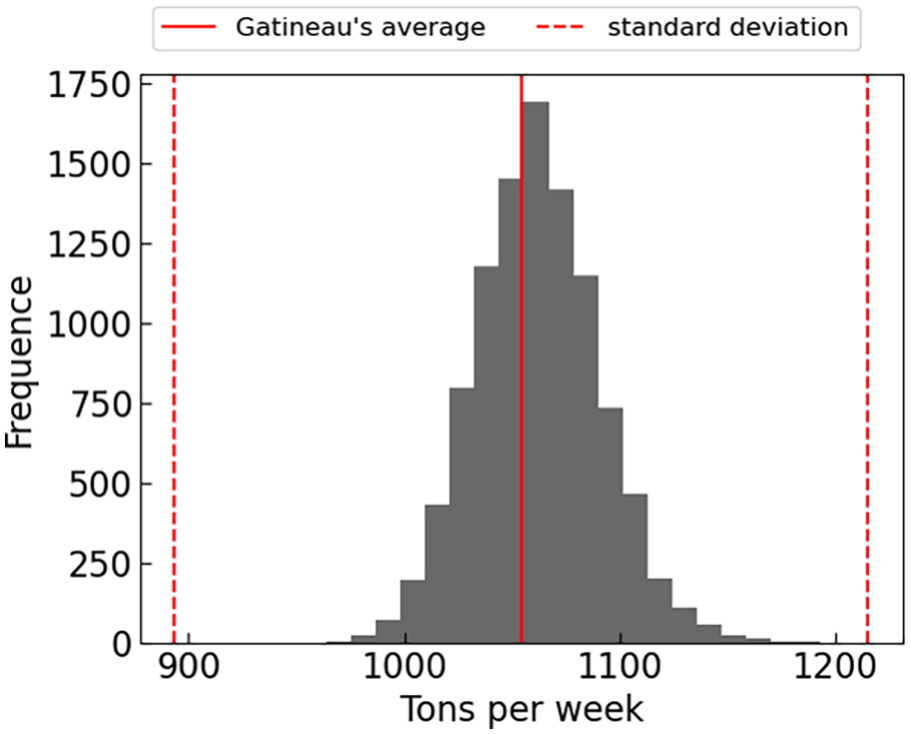
Mixed waste production predicted per week compared to Gatineau’s official reported generation data.

Following the calibration, simulations were conducted based on the entire population of Gatineau. The simulated generated weight was compared to the actual weights produced each week on the territory for the specified time interval. For the simulations, each household agent in the territory was assigned a random value of mixed waste generation rate per week from the distribution specific to its housing type. The overall predicted generation rate distribution for Gatineau is depicted in Figure 12. The simulation results indicate an average close to that of the actual Gatineau’s distribution with a difference of 0.96%. However, the actual standard deviation, represented by the dotted values for Gatineau’s data, is higher than the predicted one with a difference of over 84%. This suggests that the simulation results predict a much tighter generation rate distribution, indicating precision in value with less accuracy in variability.

Two key statistical measures, skewness and kurtosis, were computed to better characterize the nature of this distribution. Skewness, which quantifies the asymmetry of the distribution, was found to be 0.072. The skewness value is close to zero, indicating a relatively symmetrical distribution (Shanmugam and Chattamvelli, 2015). In the context of waste generation, this suggests that there is a tendency for some instances to exhibit higher waste generation rates than the average. Additionally, we calculated the kurtosis of the distribution, yielding a value of 0.039. This kurtosis value, indicates that the distribution is platykurtic, meaning it is less peaked and has thinner tails than a normal distribution. This implies that most waste generation rates are relatively close to the mean, with fewer extreme values and that the waste generation rates are generally consistent.

**Figure 13.**
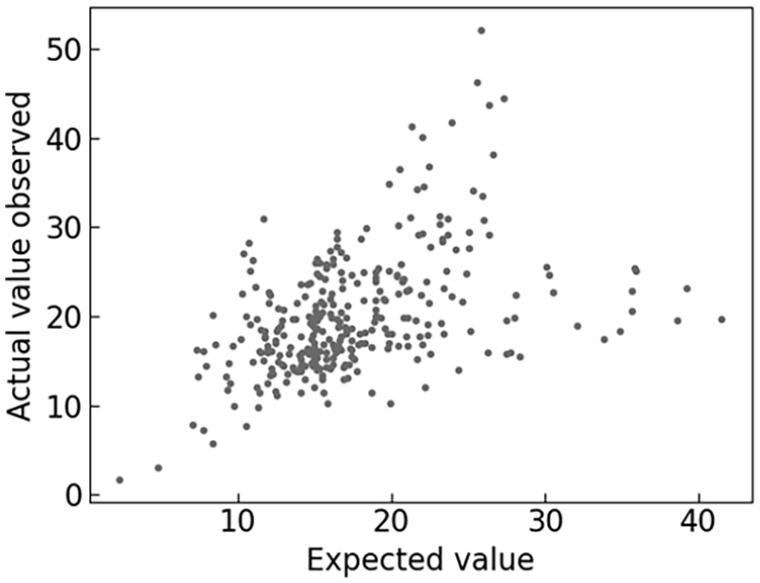
Comparison between actual value observed and the expected value for the test dataset.

**Figure 14.**
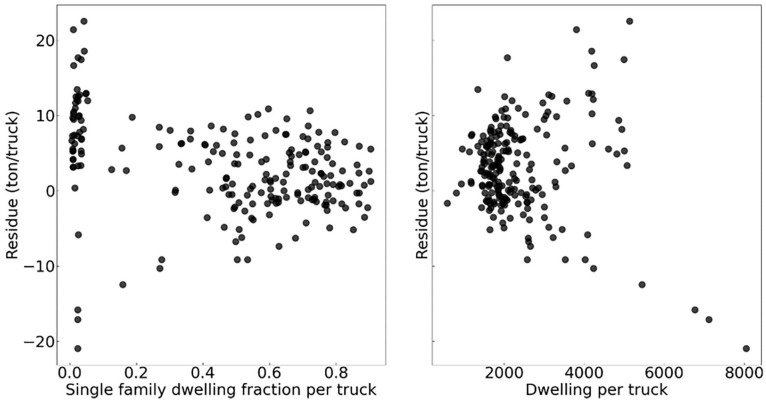
Residue per truck for the test dataset.

Analysing the residues from each truck data in the test set (Figure 13) makes it possible to observe heteroscedasticity in the results. The error term is not constant between observations and increases with $Y_{i}$ (tonnes/truck/day). The presence of outliers or omitted variables in the model is the main cause of this phenomenon. Although on a macroscopic scale, it is possible to correctly represent the population, in this model, too many parameters are neglected to provide an accurate representation of a single truck or a single household. This seems to be more apparent for multi-dwellings as observed in Figure 14. The model should therefore be used on a population slightly larger.

## Discussion

In the field of waste management and end-of-life product flow strategies, the significance of real-time geolocalized data cannot be overstated. Our work marks a significant advancement in utilizing such data by establishing a standardized framework. This framework’s primary objective is to rectify the inherent biases that can infiltrate the process of data collection and analysis, a critical step in overcoming challenges exposed by Dias et al. (2021). By implementing this methodology, we have contributed to a more accurate representation of MSW generation, particularly in terms of a potential weekly value distribution. The next three sections present the general ideas provided by the framework as well as the limitations of the resulting predicted model.

### Setting the data environment for a proper model

When creating a MSW generation model, it is important to consider various data preprocessing techniques, such as data selection, data separation (training/test split) and outlier removal, to ensure model accuracy and data environment adaptability. Knowing the large uncertainty and variability in available data, our approach was to perform sensibility analysis on those preprocessing techniques in order to select those with the most limited negative impacts on the model accuracy and precision.

For example, the training/test split value selection is a common technique used in machine learning to evaluate the performance of a model with unseen data. By dividing the dataset into training and test subsets, we aim to strike a balance between ensuring that the model learns from the data without overfitting to it and verifying its ability to generalize to new, unseen data. The standard split is typically 70/30 or 80/20, with most of the data used for training (Brownlee, 2016). However, it is important to note that the choice of the split ratio is not one-size-fits-all. The ideal split ratio can vary depending on factors such as the dataset size and complexity, as well as the specific problem being addressed (Muraina, 2022). In our case, the framework we employed revealed the necessity of adjusting the split ratio, as indicated by the presence of overfitting (Figure 7) when the standard split values were used. This underscores the importance of tailoring the split to suit the unique needs and characteristics of the dataset and machine learning task.

Moreover, in the context of an MSW generation predictive model, outliers may represent unusual waste generation patterns that are not necessarily representative of the population as a whole. Removing outliers can improve the accuracy of the model by reducing the impact of these unusual data points. However, it is important to carefully consider the criteria used to identify outliers and to ensure that the removed data points do not represent significant information. Looking at discarded data profile (Figure 9) is mandatory to ensure no distinct population is targeted.

### Housing as a key modelling parameter

The results of our study highlighted the significance of housing types as a critical socioeconomic and demographic parameter for accurately predicting MSW generation rates. However, our findings diverged from certain prior studies, such as (Lagneau, 2018), as we observed that the inclusion of duplexes in our model did not lead to an enhancement in its predictive accuracy. With a larger surface area than a traditional apartment, a duplex apartment allows citizens to enjoy the benefits of city life, while adopting some of the characteristics of single-family dwellings. Their profile can therefore be sometimes closer to either multi-family dwellings or single-family dwellings.

Several studies (Abbott, Nandeibam and O’Shea, 2011; Ferrara and Missios, 2005, 2012; Huang et al., 2011) deplore the fact that neglecting the effect of citizen behaviour is detrimental to the use of the models and the understanding of the studied phenomena. Decision support tools must therefore consider the heterogeneity of the population in terms of sociodemographic profile and household behaviour.

Similarly, bin size is a key parameter for recycling efficiency. In the event of a full container, only 55% of individuals deferred keeping the material until the next collection (Schilling, 2001). An increase in the size of recycling bins is known to increase recycling participation and diversion rates (Lane and Wagner, 2013). Sharing a bin or container with other households may influence material sorting by the citizen and thus the amount of material going to landfill. This reinforces the idea that household behaviour in response to municipal incentives, as well as their socioeconomic ownership, has some impact on the total amount of material generated by a municipality and should be included in this type of study.

To accurately represent duplexes as housing units with distinctive waste generation behaviour in the context of Gatineau, it may be necessary to include additional socioeconomic properties in the description of agents. This approach recognizes that the characteristics and waste generation patterns of dwellings are not universally transferable across different regions, and regional-specific factors play a significant role in shaping waste generation dynamics. Consequently, our study underscores the importance of tailoring predictive models to account for unique regional characteristics, thereby enhancing their accuracy and applicability in specific geographic areas.

Moreover, the observed tightness in the mixed waste production simulation, signifying precision with reduced variability, is a notable outcome. Although the simulation captured the average waste production of Gatineau well, the higher standard deviation in the actual data highlights the complexity and variability inherent in waste generation. Over the course of the year, weekly waste production fluctuates based on seasonal changes (Mendes et al., 2013), consumption patterns (Zacarias-Farah and Geyer-Allély, 2003) and holidays (Whitmarsh et al., 2018). These external factors contribute to the observed variability in actual waste generation rates, which the current simulation, with its tighter distribution, may not fully encapsulate. Incorporating these external parameters into the simulation model could enhance its capacity to mirror real-world variability in waste generation, providing a more comprehensive understanding and prediction of waste patterns. Therefore, further refinement of the model by introducing consumption-related parameters could contribute to achieving a more realistic representation of waste generation patterns and addressing the observed differences in standard deviations.

### Predictive model limitations

Waste generation is inherently uncertain and can be affected by factors that are difficult to predict, such as changes in consumption patterns or technological advances. External factors, such as climate change, environmental regulations or economic incentives, can also significantly affect waste generation patterns. Although these factors are challenging to incorporate into a model, they play a pivotal role in explaining why waste generation patterns vary considerably across different regions. It’ is important to note that the results reported in this study are specific to the region of Gatineau, where the data were collected. However, the model framework developed here may offer valuable insights and serve as a foundation for similar studies in other regions or for different waste types, with the necessary adjustments to account for regional variations and specific waste characteristics.

Moreover, any model in MSW will be limited by the lack of accurate and up-to-date data, which can make it difficult to accurately predict future waste generation.

The lack of an RFID chip in a waste generation model limits the accuracy and effectiveness of the model, reducing its ability to inform waste reduction strategies and improve waste management systems. We used a GIS artefact to overcome this limitation. However, by doing so the accuracy of the model is impacted and a sensible parameter is introduced to the model. This can affect the foundation of subsequent work but can answer municipalities perceived requirements in terms of accuracy and precision.

## Conclusion and future work

In this study, we have presented a comprehensive framework that addresses critical challenges in the field of waste management, specifically pertaining to the prediction of MSW generation. The utilization of real-time geolocalized data offers an opportunity to enhance MSW generation modeling and inform sustainable waste management strategies. By presenting an adaptable framework, complemented by the agent-based model construction and its series of sensitivity analyses, our research poses the foundations for the development of highly sought prediction models of end-of-life product flows. Considering the large-scale implications and impacts of waste management and end-of-life product flow strategies, it is critical to conduct thorough analyses. Failure to do so may lead to inefficient and wasteful strategies, not adapted to evolving waste generation behaviours.

Although this research focuses on mixed waste in the city of Gatineau, the conceptual and methodological foundations laid down serve as a blueprint for similar studies, offering a versatile and scalable solution for assessing waste generation dynamics across diverse contexts. Waste collection routes that are non-uniform can pose substantial difficulties in model calibration. To effectively harness the power of geolocalized data, our study employed a comprehensive data transformation and hybridation process, coupled with innovative buffer integration. We tailored a reverse geocoding algorithm using buffers, enabling accurate dwelling-to-truck assignments. This flexibility is instrumental in ensuring that the framework can accommodate variations in data volume, ranging from a few collection trucks within a single city to the complexities of data collection from multiple municipalities, thus meeting the practical needs of waste management systems.

Future research will expand upon this foundation by using the framework to accommodate multiple collection types, making it adaptable to various waste management scenarios. Additionally, future work will delve into the behavioural aspects of dwellings within the waste generation process, particularly in the context of sorting decisions influenced by environmental attitudes. By connecting the predicted waste generation rates with dwellings’ recycling intentions in an agent-based model, we aim to provide municipalities with valuable insights for estimating waste quantity and quality, as well as evaluating the environmental impact of their waste management strategies. These endeavours represent a step forward in advancing the sustainability and efficiency of waste management systems.
